# Traditional Meat Products—A Mycotoxicological Review

**DOI:** 10.3390/life13112211

**Published:** 2023-11-14

**Authors:** Krešimir Mastanjević, Dragan Kovačević, Ksenija Nešić, Vinko Krstanović, Kristina Habschied

**Affiliations:** 1 Faculty of Food Technology, Josip Juraj Strossmayer University of Osijek, 31000 Osijek, Croatia; kmastanj@ptfos.hr (K.M.); dkova@ptfos.hr (D.K.); vkrstano@ptfos.hr (V.K.); 2 Food and Feed Department, Institute of Veterinary Medicine of Serbia, Smolućska 11, 11070 Beograd, Serbia;ksenija.nesic@gmail.com

**Keywords:** dry sausage, cured meat products, mycotoxins, traditional production, fungi

## Abstract

Traditional meat products are commonly produced in small family businesses. However, big industries are also involved in the production of this kind of product, especially since a growing number of consumers crave the traditional taste and aromas. The popularization of original and organic products has resulted in a return to traditional production methods. Traditional meat products are produced worldwide. However, in such (domesticated) conditions there is a potential danger for mycotoxin contamination. This review aims to present the sources of mycotoxins in traditional meat products, the most common mycotoxins related to such meat products, and future prospects regarding the suppression of their occurrence. Special attention should be paid to reducing the transfer of mycotoxins via the food chain from animal feed to animals to humans (stable-to-table principle), which is also described in this review. Other sources of mycotoxins (spices, environment, etc.) should also be monitored for mycotoxins in traditional production. The importance of monitoring and regulating mycotoxins in meat products, especially in traditional meat products, is slowly being recognized by the institutions and hopefully, in the future, can deliver legally regulated limits for such products. This is especially important since meat products are available to the general population and can seriously affect human health.

## 1. Introduction

Traditional meat products mostly refers to dry-cured meat products produced in a traditional manner. The majority of meat industries try to imitate the traditional production, but such products always show a certain discrepancy in aroma and texture when compared to traditionally produced cured meat products. However, the demand for high-quality and health-safe products enables many small producers to step in the market with traditional meat products. Different dry-cured meat products require high quality meat and production conditions that satisfy basic health and hygienic standards [1].

However, the nature of the production of dry-cured meat products often involves the use of (starter) cultures of molds on the surface of dry-cured products such as dry-cured hams, prosciuttos, and sausages (Kulen). Starter cultures of bacteria such as *Staphylococcus xylosus* and lactic acid bacteria can be added to meat products as well. The direct contact of the meat product with molds could result in mycotoxicological contamination, as confirmed by many authors [2,3,4,5,6,7,8,9]. Potential sources of contamination during meat processing and cured meat product production are present in all stages of manufacture. Spices are one source and the environment in which the manufacturing is conducted is also a potential source of mycotoxins; however, the long ripening stage is the point at which mycotoxins have the highest potential to occur since this phase involves higher substrate moisture, high air humidity, and temperatures favorable for fungal growth. 

Mycotoxins represent health hazard to humans and animals, and as such they can be carried over from “stable to table”. Their concentrations in different foodstuffs have been limited by legislation, including cereals and animal feed. However, meat products are yet to be designated maximal allowed amounts of mycotoxins. So far, only OTA (ochratoxin A) has been under consideration by the EFSA’s (European Food Safety Agency) scientific opinions [10]. The toxic effects of mycotoxins have been described in many papers and mostly involve carcinogenic, teratogenic, neurotoxic, hepatotoxic, and nephrotoxic effects. In addition, they can cause immune toxicity, reproductive and developmental toxicity, and many other health-related problems [11,12,13], and thus should be regularly monitored and regulated by legislation.

The aim of this study is to give an overview of known mycotoxins that can be found in different traditional dry-cured meat products and to present the major molds that can be found on such products. Describing the possible sources of mycotoxins contamination and presenting the possible reduction methods regarding the “from stable to table” principle, gives additional value to this review. The cross-section of possible methods for the reduction of mycotoxins in animal feed can contribute to the overall awareness of mycotoxins. This review aimed to report the possible sources of contamination, potential and commonly found mycotoxins in different traditional meat products, transfer routs from “table-to-stable”, possible reduction and prevention methods and strategies, and future perspective regarding legislation and monitoring.

## 2. Traditional Dry-Cured Products—Common Fungi

**Figure 1:**
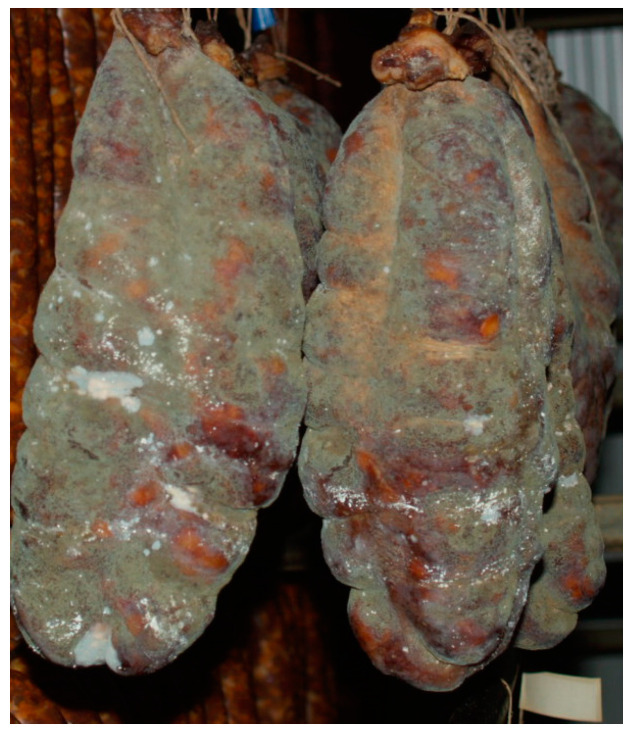
Molds on the traditional Croatian dry-cured sausage Slavonski Kulen.

**Figure 2:**
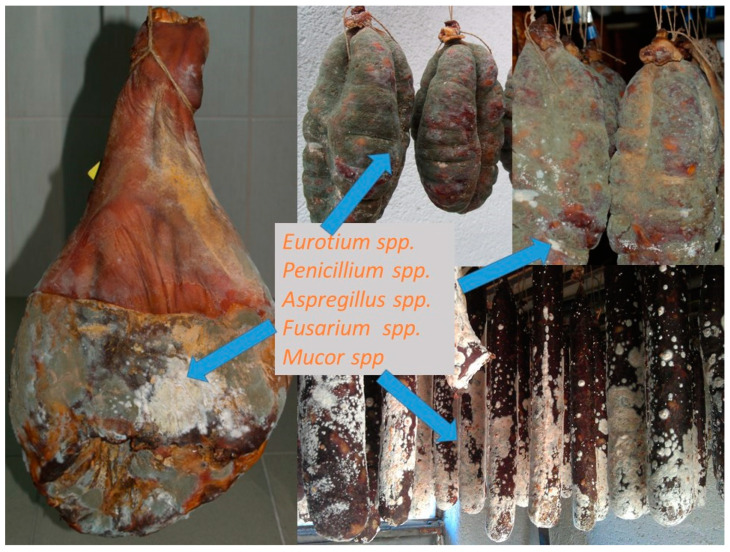
Different molds on traditional dry sausages and dry-cured meat products.

Traditional dry-cured meat products are indigenous to many countries, especially in Europe where molds play an important role in the ripening stage of production (Figure 1). Italy, Spain, France, Hungary, Croatia, and Southern Germany traditionally use white and occasionally green mold cover on the surface of dry-cured products. They are greatly appreciated due to the development of a characteristic taste, flavor, texture, and appearance of dry-cured meat products. The color of the mold cover depends on the species, but often it is influenced by the temperature during the ripening phase. For example, growth above 15 °C stimulated green conidia formation [14]. This is particular for traditional manufacture since they usually do not have controlled humidity and temperature conditions and depend on environmental conditions, thus the resulting molds can sometimes be white and sometimes greenish. Common molds used in some traditional dry-cured meat products are filamentous fungi such as *Alternaria*, *Aspergillus*, *Cladosporium*, *Eurotium*, *Mucor*, *Penicillium*, *Rhizopus*, and *Scopulariopsis*, as reported in the studies focused on fermented sausages and dry-cured hams [15,16,17,18,19,20,21,22,23,24,25,26,27,28,29,30,31,32,33,34,35,36,37,38,39,40,41,42,43], as shown in Figure 2. *Penicillium* was detected and identified in some samples of traditionally produced fermented sausages and only in several samples of dry-cured ham [4,15,17,24,27,31]. In some samples it was identified together with *Scopulariopsis* [21], *Aspergillus* [16,20], and *Eurotium* [6]. 

According to several sources, *Aspergillus* and *Eurotium* have been shown to be the prevailing molds on dry-cured hams [4,24,26,28,41,42]. Molds belonging to genera *Eurotium* are xerophilic and prosper on substrates such as dry-cured hams, with surfaces with low water activity (<0.80) [32]. Fermented sausages were commonly contaminated with *Penicillium* spp. Similarly to *Eurotium* spp., *Penicillium* species tolerate low water activity (0.78–0.83) and protein-rich substrates. They proliferate in lower to mid temperatures [42,43,44]. Some species belonging to *Penicillium* genera, such as *Penicillium nalgiovense* have been used as mold starter cultures for the industrial production of mold-fermented sausage [14,43]. *Penicillium nordicum*, as an ochratoxin A producing species, was shown to have a significant share in the studies where it was reported [4,6]. 

The most common fungi and mycotoxins detected on different meat products are shown in Table 1. As can be seen, different fungal species can be found on meat products. Various mycotoxins can be produced by fungi, but some can be found in combination, which is called co-occurrence. This is an especially health-concerning topic for scholars since multiple toxins can be detrimental to human health with a combination of side-effects.

Environmental contamination of meat products can occur via conidia, ascospores, or mycelium fragments, but not many of them can grow on meat products, especially dry-cured ones [42]. According to [29] *Cladosporium* was found to be the dominating contaminant in the fermented sausages.

## 3. Mycotoxins in Dry Sausages and Dry-Cured Meat Products

Mycotoxins can end up in dry-cured products through different pathways. Most commonly they end up in animals via contaminated feed. The addition of spices in the meat and/or stuffing can also contribute to the contamination. However, many scientific investigations have aimed to clarify the origin of mycotoxins in dry-cured meat products and relied on the thesis that molds growing on the surface of the meat product can be the source of contamination. Even though EU legislation does not yet include mycotoxins in meat and meat products, OTA has currently been under consideration by the EFSA’s scientific opinions. More will be described in the following sections. 

Mycotoxins enter the food supply chain and end up in markets which can be detrimental to human health. There are two ways for mycotoxins to enter the food and feed chain: direct and indirect [46,47]. Meat as a raw material can be contaminated via the feed used for animal feeding (carry-over effects), and consequently, can indirectly contribute to human exposure. In addition, spices used for meat products’ production are reportedly a potential direct source of mycotoxins [48,49]. Comi and Iacumin [50] reported OTA in hams, probably as a result of direct contamination with molds. This can also be explained by indirect contamination as described by several authors [51,52,53]. Similarly, OTA can be found in sausages. The pathway of contamination can be identified as indirect transmission via pigs’ feed and by direct contamination via molds which can grow on raw meat after slaughter [54,55]. The environment in which the production is carried out can also be a source of fungi that can proliferate in meat products during the ripening phase. This phase is particularly important since it involves high humidity and favorable temperatures for fungal growth. 

The production of traditional meat products, especially dry sausages, relies on the addition of different spices to deliver the familiar aromas and taste. The most used spices are pepper (white, red, and black), sweet and spicy ground paprika, and garlic. Certain traditional products can be spiced with laurel and rosemary [56]. Despite the antifungal activity that many of these spices display, they can be contaminated with molds as well [57]. Common contaminants found in spices belong to *Aspergillus* and *Penicillium* genera [58,59,60,61,62]. Due to poor production conditions (drying spices on the ground) spices such as chili, nutmeg, and paprika powders can contain aflatoxins (AFs), OTA, and certain different mycotoxins. Some samples even exceed the maximum EU legislative limits. Spices sold at farmers markets can even have significantly higher mycotoxin concentrations than those bought at supermarkets [56,63,64]. Spices such as paprika and black pepper can contain significant amounts of AFs and OTA [65,66,67,68,69]. According to Gambacorta et al. [65], AFB1 concentrations in paprika can reach 155.7 µg/kg, and in black pepper it can be up to 75.8 µg/kg [66]. High values of OTA can also be found in paprika, amounting to 177.4 µg/kg [65] and 79.0 µg/kg in black pepper [67]. A higher prevalence of OTA in prosciutto samples was sometimes linked to pepper spiking; namely, pepper often becomes contaminated with *Aspergillus* molds, out of which *A. niger* produces OTA [20,60]. However, some studies have revealed that spices may also inhibit mold growth [61], resulting, for instance, in lower OTA contamination in some meat products [20].

OTA can also be found in certain prosciutto samples due to pepper used for coating, since molds such as *Aspergillus* spp. commonly contaminate pepper [70]. OTA can be found in dry-cured hams, as well. However, according to the literature, this contamination probably occurs during ripening, due to direct contamination with molds, presumably because of the inadequate environmental conditions, i.e., increased air humidity and higher temperatures [2,3,4,7]. 

**Figure 3:**
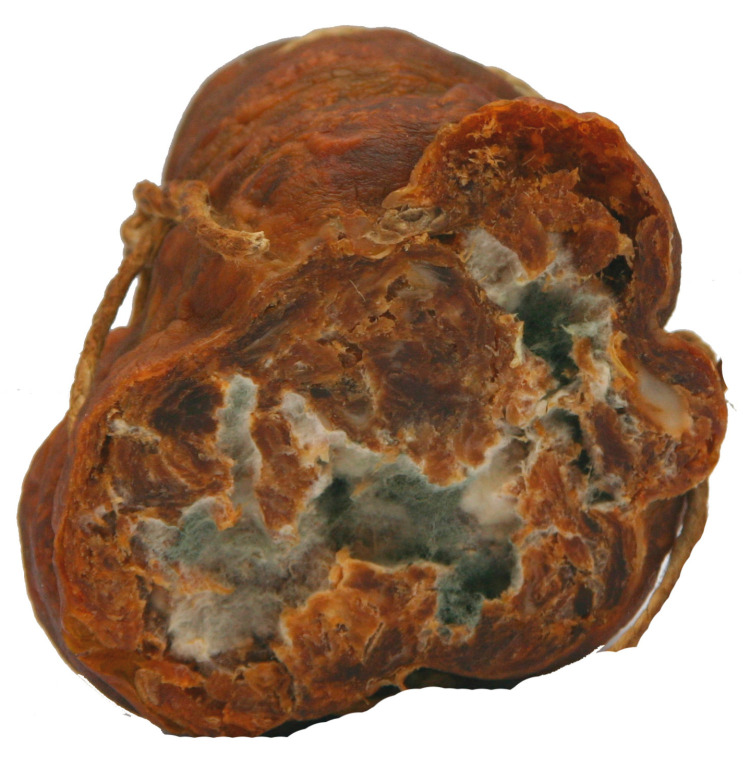
Molds inside a dry sausage due to damaged casing.

Sometimes, damages to the outer casing can enable the entrance of molds and mycotoxins into sausages (stuffing) (Figure 3), and in significant amounts, according to [7,70,71]. 

However, Iacumin et al. [6] reported OTA contamination of the casings (outer layer) while the inner layer was not contaminated. According to [8] this can also present health hazard since casings are usually sliced together and consumed with the stuffing. 

OTA is a natural contaminant in different foodstuff and is often detected in meat products. *Aspergillus ochraceus* and *Penicillium verrucosum* are the most common producers of OTA concerning the meat industry. Direct contamination usually occurs via animal feed [72,73,74]. OTA is fat-soluble and can mostly be found in the kidney, lung, liver, blood, spleen, heart, and adipose tissue of pigs [74,75]. A study conducted by Perši et al. [48] showed that pigs who were given 300 μg/kg/day of OTA for 30 days, accumulated it in the kidneys, lungs, and fat tissue. OTA was then detected in different meat products such as blood sausages, liver sausage, and pâté [76]. Even though OTA is not legally regulated in the EU, some countries have recognized the importance of strict limits for this mycotoxin and have set the limit to 5 μg/kg in pig liver, kidneys, and meat (Romania). In Italy the limit is much lower, set at 1 μg/kg in pig meat and meat products [76]. Suppression of OTA in meat products can be carried out by proper prevention via food safety management systems. In cases where prevention is not sufficient, then a set of diverse methods of physical and chemical treatments can be applied in order to reduce the contamination. However, chemicals used for decontamination can impair the sensory properties of dry-cured products which can drive the customers away. Much research has been devoted to finding a biological prevention method, and so far, essential oils have been meticulously investigated for this purpose. Even though essential oils can significantly affect the sensory properties of such products, the involvement of novel encapsulation technologies could help reduce such changes [77]. So far, oregano, garlic, sage, peppermint, rosemary, neem, and eucalyptus have been identified as being successful in suppressing mold growth and OTA production [78,79,80].

Aflatoxins are commonly produced by *Aspergillus* spp. (*A. flavus* and *A. parasiticus*). Aflatoxin B_1_ (AFB_1_) is known for its high potential for carcinogenic and genotoxic properties. Its metabolite is AFM_1_, an aflatoxin that can be found in milk, where it ends up through ingestion of contaminated feed. AFB_1_ is not often determined in meat foods, and when it is, its concentrations are much lower than OTA. It can also be found in different tissues such as liver, muscle, and fat tissue [81]. According to the IARC (International Agency for Research on Cancer) it belongs to Group 1 (human carcinogen) and is associated with the occurrence of liver cancer [82,83]. Even though AFB_1_ is not as common as OTA, it can still be found in different processed meat products [84]. Similarly, like OTA, AFs can be efficiently suppressed by using essential oils. As an effective agent, onion has been designated as an effective AF inhibitor in the meat industry [85]. Saffron, Shirazi thyme, estragon, basil, black cumin, coriander, dill seeds, and Arabian incense can suppress AF production [86,87,88,89,90,91,92,93,94].

Zearalenone (ZEA) is designated as the oestrogenic mycotoxin, according to the IARC it is described as a Group 3 carcinogen. ZEA causes hormonal disbalance related to cervical, ovarian, and prostate cancer [95,96]. Its derivate, α-zeranol, can be used as a cattle growth agent, but so far, the EU has not approved this [77]. ZEA is commonly found in chicken meat [97], sheep meat, and beef meat [98]. To reduce ZEA levels lemon, grapefruit, eucalyptus, and palmarosa essential oils were investigated, but the activity of these oils cannot be interpreted as being significantly effective [99].

According to some authors, citrinin (CIT) can be found in dry-cured traditional meat products as well [100]. Citrinin displays hepatic and nephrotoxic effects, and is generally produced by *Penicillium* spp., but specifically by *Penicillium citrinum*. *Aspergillus*, and *Monascus* genera can also synthesize this mycotoxin, originally named monascidin. CIT can be found in kidneys, causing renal degeneration associated with weight loss [101,102]. According to the IARC it belongs to Group 3 [103]. Even though it can be found in different meats and meat products including dry-cured meat products [100,104,105], there is a minimal contribution to increased CIT intake in humans, given the low rate of CIT transfer from feed to tissue for consumption [106,107].

Patulin (PAT) is mycotoxin synthesized by several species belonging to genera *Penicillium*, *Aspergillus*, and *Byssochlamys*. It displays toxigenic properties [108]. PAT has carcinogenic potential, being classified as being in Group 3 by the IARC as well [109]. In meat products PAT usually co-appears with other mycotoxins. PAT and OTA were detected in dry-cured hams [110]. Since PAT is not incidental, very few studies have been conducted regarding the usage of essential oils in patulin suppression in meat products [77]. 

Sterigmatocystin (STC) is in Group 2B, according to the IARC [111]. It can be found in pork muscle [112]. 

Fusarenon-X (FX) is designated as a trichothecene belonging to group B, and the IARC classified it as Group 3. It can often be found in food and feed. In livers and kidneys, it can be converted to nivalenol. The IARC has classified these toxins as belonging to Group 3 [113,114]. 

T-2 toxin is often found in cereals and cereal based products and is a metabolic product from *Fusarium*, *Myrothecium*, and *Stachybotrys* genera [115]. T-2 was detected in back muscle, pig back fat, and chicken muscle in concentrations less than 0.5 μg/kg [75].

Deoxynivalenol (DON) causes acute emesis, gastroenteritis, diarrhea, and reduced food consumption with chronic implications. It can be found in pig back fat, muscles, and liver [11,116].

Cyclopiazonic acid (CPA) is characterized as a dangerous mycotoxin that can cause damage to the digestive organs, the myocardium, and the skeletal muscles, and cause neurological disorders. Its producers belong to *Penicillium* and *Aspergillus* spp., specifically *Penicillium commune* [33,34,35] which was isolated from the surfaces of different meat products, including European dry-fermented sausages and prosciuttos [17,24,36,117,118,119].

## 4. Prevention Methods

The reduction or suppression of mycotoxins in meat products is possible through the prevention of the proliferation of toxigenic fungi [36,120,121,122,123,124] (Table 2). This can be achieved by applying different chemical preservatives or by utilizing packaging in a modified atmosphere. The stated methods are not suitable for dry-cured meat products since completely halting microorganism activity would affect the sensory properties, and most importantly, traditional dry-cured meat products are desired among population for their chemical-free status [36,125,126,127,128,129,130]. 

Heating, salting, drying, and storage were shown to be inefficient in reducing, e.g., OTA concentrations in the final product, since OTA is a stable molecule, and resists high temperatures and fermentation which enables OTA contamination of the final meat product [48,70,131]. One possibility to reduce the mycotoxins contamination is to monitor and regulate the a_w_ of the substrate since it affects the fungal ability to produce mycotoxins. Thus, it is possible to suppress the production and accumulation of mycotoxins by controlling the a_w_ level and temperatures during the drying and ripening stages. For example, *Penicillium polonicum* synthesized significantly higher verrucosidine levels at a_w_ of 0.99 compared to a_w_ of 0.97 and 0.95 [1]. Both physical and chemical decontamination options display certain limitations, the most important being the loss of nutritional value and modified sensory characteristics, while biological means of decontamination were shown to be a positive option for reducing mycotoxins or even preventing them from entering the human metabolism [132]. 

The application of antagonistic microorganisms is also a good approach to replace the chemical and physical methods [11]. One of these methods is the utilization of indigenous yeast and fungi as dry-cured meat product preservatives. Some authors reported this as a useful way to prevent the proliferation of ochratoxigenic fungi [133,134,135,136]. This is an effective method since it relies on competition for nutrients and space, and microorganisms synthesize chemicals which are active against undesirable fungi, i.e., antifungal proteins [130]. The most common antifungal proteins isolated from fungi are shown in Table 3: 

Treating meat products with ozone suppresses the proliferation of fungi known to produce mycotoxins [132]. 

Gamma radiation is also reportedly successful in suppressing mycotoxin-producing fungi, but its success depends on several factors such as the number and type of fungal pedigree, dose, food composition, and air humidity [132,138,139]. 

Meticulous physical removal of molds from the surface of the product during ripening stage can also be helpful. This could be performed by brushing or by washing in order to remove the visible patches of molds [1,120]. According to [6], in order to lower OTA contamination in sausages, they should be brushed and then washed. 

However, it is important to act during the ripening stage, to prevent the molds from proliferating on the surface. This could easily be carried out by ensuring the proper distance between the products to enable the sufficient air flow between them. If maturation is conducted in chambers, the equipment should contain biological microfilters which enable fresh air supply. For additional safeguarding, chambers should be sprayed with fungicidal coatings, and the entrance should have a pressure barrier [140]. 

Most important is the reduction mycotoxin levels in all steps of production, “from stable to table”. Extremely important is the control and reduction of the carry-over effect, meaning that animal feed should be strictly monitored for mycotoxins. This should ensure the mycotoxin-free or at least minimal levels of mycotoxins in raw materials for meat products. In case mycotoxins do enter the food chain and end up in animals, detoxification methods should be conducted [141]. 

Another way to prevent the carry-over of mycotoxins to meat products is to reduce the addition of fungal derivates commonly used as pigments, which can be contaminated with mycotoxins, concretely with citrinin, as reported by [142]. This extract is obtained from red mold rice (*Monascus* extract), illegal in the EU, but still can be found in some products [142].

The basic action for reduction of the carry-over effect is the application certain control strategies. Firstly, fungal microflora should be suppressed in the field. Using the proper agro-technical measures, mycotoxin production could be controlled prior to the production of feed. Secondly, maintenance of the grains’ integrity is of the utmost importance. Appropriate water content, oxygen concentration, and temperature during storage of grains and animal feed can significantly suppress mycotoxin production. The application of antifungal chemicals such as propionic acid, sodium chloride, and ammonia is useful in fighting fungal contamination [142]. 

Mycotoxins in animal feed can be reduced in several ways [143,144]:

1. Physical methods:
  - Washing grain with water or sodium carbonate;
  - Manual sorting of contaminated grains based on the physical aspect of grains or using fluorescence to detect the presence of mycotoxins;
  - Exposure to high temperatures, UV, X-rays or microwave irradiation;
  - Solvent extraction of toxins;
  - Dilution of contaminated feed with non-infected feed;
  - Supplementation of binding agents which bind mycotoxins in order to decrease the bioavailability of these compounds in animals; hydrated sodium calcium aluminosilicates (HSCAS) and phyllosilicates derived from natural zeolites have a high affinity to AFB_1_; zeolites (hydrated aluminosilicates of alkaline cations), bind with AFB1 and ZEN [145,146]; bentonites bind with AFB1 and T-2 [147]. Kaolin, sepiolite, activated carbon and montmorillonite bind AFB1. Activated carbon is obtained by pyrolysis and the activation of organic compounds. It has a more heterogeneous porous structure. Activated carbon is also able to bind mycotoxins [148]. Resins such as cholestyramine and polyvinylpolypyrrolidoxynivalenol are also able to bind OTA and AFB1 [146]. However, all of them show an adverse effect to bioavailability of minerals and vitamins. Namely, they reduce the bioavailability of vitamins and minerals, and some of them are potential heavy-metals and dioxin carriers [149].
2. Chemicals: acids, bases (ammonia, caustic soda), oxidants (hydrogen peroxide, ozone), reducing agents (bisulphites), chlorinated agents, and formaldehyde have been reportedly successful in degradation of mycotoxins [143].
3. Microbiological methods: lactic acid bacteria, propionibacteria, and bifidobacteria cell wall structure are efficient in binding mycotoxins [150,151]. Mycotoxins are then eliminated in the feces without significant detrimental effects on the animals or any risk for toxic residues to be found in edible animal products. Glucomannans found in the cell wall of *Saccharomyces cerevisiae* bind to AFs’, FUM, ZEN, T-2, CIT, DAS, DON, OTA, NIV, and fusariotoxin. *Corynebacterium rubrum* can biotransform mycotoxins in contaminated feed [152].

The reduction of mycotoxins in dry-cured meat products usually requires the use of fungal-species starter cultures designated as GRAS (generally regarded as safe). The selection is based on bioassays. All starter cultures used in the production of sausages (for example, Italian dry sausages) have to be investigated for their technological safety, they must not produce mycotoxins, and have to provide suitable technological properties regarding aroma and taste. Additionally, they have to provide a compact layer on the surface of the product. Mostly, such molds belong to genus *Penicillium* [142]. 

## 5. Future Prospects and Legislation

Fungal contamination and mycotoxin occurrence are tightly related. It is impossible to avoid fungi during the production of dry-cured meat products. This is because fungi are ubiquitous and can be found on clothes, in spaces, and on the equipment of the producers. This is especially prominent in traditional production due to the lack of microbiological filters and pneumatic barriers. This affects the temperature and relative air humidity, leaving it uncontrolled and unmonitored. During ripening, many products are covered with molds. Usually these spores are indigenous to the producer’s ripening chamber. Prolongation of ripening commonly amplifies the mold coverage which can enhance the mycotoxins coverage [81]. Considering the dangers of food contamination via mycotoxins, and knowing the harmful effects they can have to human health, some mycotoxins are involved in the legislation in order to reduce their concentrations in foodstuffs. However, they are not yet regulated in meat products, at least at the EU level. Mycotoxin limits for food products are generally unified with the EU legislation. However, mycotoxins in meat products are still not included within the EU’s legislation and are variable for every country. Certain countries do not control or even have mandatory hygienic standards for meat products despite the significant risk of mycotoxin contamination [153]. 

Currently, only AFs and OTA have been under regulations in food of animal origin (e.g., milk and milk products), but meat and meat products are not included. This is based on the safety protocols regarding toxins that can be found in foods of vegetable origin. Even though several mycotoxins are regulated, with the recommended values being based on the knowledge of toxicity and potential accumulation of these molecules in animal derivatives, novel regulation regarding meat and meat products should be considered [153]. Some EU countries, such as Italy, Denmark, Estonia, Romania, and Slovakia, have prescribed mycotoxin limits for meat and meat products. For example, the Italian Ministry of Health has prescribed a maximum value of 1 μg/kg for OTA in pigs’ meat or meat products [6,76,129]. Other countries that have introduced mycotoxins into their legislation are Denmark (10 µg/kg in pig kidney), Estonia (10 µg/kg in pig liver), Romania (5 µg/kg in pig kidney, liver, and meat), and Slovakia (5 µg/kg in meat) [76]. The American, Asian, and African continents, and other European countries, have not jet regulated the maximal permitted levels of OTA or AFs in meat products [46]. 

The implementation of maximal limits for mycotoxins that can be found in dry-cured meat products would significantly aid the reduction of mycotoxin intake in humans and would surely improve the quality of such products. Since many consumers appreciate the traditional approach, smaller family producers should work on improving the working conditions (equipment, sanitation, monitoring of mold spores in ripening chambers, etc.) during the production of dry-cured meat products. 

Table 4 shows the overall presence of common mycotoxins in meat and meat products, their concentrations in meat and meat products and related to a regulatory body for potential (future) legislation. Some mycotoxins, such as OTA, aflatoxin B1, sterigmatocystin, zearalenone, and T2, occur commonly in raw materials, such as meat, and then subsequently end up in meat products. Aflatoxin B2 and citrinin were determined in meat products where they, presumably, ended up from unclean surfaces, spices, or other handling procedures. In any case, all of them can cause serious adverse health effects and thus should be legally regulated and continuously monitored by one of the regulatory bodies stated in Table 4.

It is clear that novel regulations will have to be introduced and will have to address not only known mycotoxins in meat and meat products, but the emerging and co-occurring mycotoxins as well (PAT and OTA [110]). This is definitely a topic for future research and legislation. 

## 6. Summary

Mycotoxins are ubiquitous in all areas of the food industry. Many industries are continuously monitoring the sources and levels of mycotoxins in raw materials and final products (baby food, cereals, etc.). However, the meat industry does not have prescribed mycotoxins which should be monitored, nor maximal allowed levels for any of them. This is important, especially since meat products are available to the general population, including children and seniors, and as can be seen from the reviewed scientific literature, mycotoxins such as OTA, AFs, CIT, PAT, DON, etc., can be found in various meat products. Uniform legislation would significantly improve the quality and safety of dry-cured meat products, but it has to involve the raw materials such as meat, spices, and even equipment used for production. In the future, in aspects of the production of dry-cured meat products, this has to be implemented.

## Figures and Tables

**Table 1:** Most common fungi and mycotoxins detected on different meat products (adopted from [45]).

Fungi	Mycotoxin
*Aspergillus* spp.	
*A. flavus*	Aflatoxin B1, cyclopiazonic acid, 3-nitropropionic acid
*A. niger*	Ochratoxin A, fumonisin B2
*A. ochraceus*	Ochratoxin A, penicillic acid, xanthomegnin, viomellein, vioxanthin
*A. versicolor*	Sterigmatocystin
*Penicillium* spp.	
*P. aurantiogriseum*	Penicillic acid, verrucosidin, terrestric acid, nephrotoxic glycopeptides
*P. brevicompactum*	Botryodiploidin
*P. chrysogenum*	Secalonic acid, PR toxin, roquefortine C
*P. citrinum*	Citrinin
*P. commune*	Cyclopiazonic acid
*P. crustosum*	Terrestric acid, penitrems, roquefortine C
*P. expansum*	Patulin, citrinin, chaetoglobosins, communesins, roquefortine C
*P. glabrum*	Citromycetin
*P. griseofulvum*	Patulin, griseofulvins, roquefortine C, cyclopiazonic acid
*P. nordicum*	Ochratoxin A, viridic acid
*P. oxalicum*	Secalonic acids, roquefortine C
*P. palitans*	Cyclopiazonic acid
*P. roquefortii*	PR toxin, roquefortine C
*P. rugulosum*	Rugulosin
*P. variabile*	Rugulosin
*P. verrucosum*	Ochratoxin A, citrinin
*P. viridicatum*	Penicillic acid, xanthoemegnins, viridic acid

**Table 2:** Common mycotoxins in dry-cured meat products.

Product	Mycotoxin	Concentration μg/kg	Country	Source
Parma (retail product)	OTA	56.0, 158.0, 113.0	Denmark	[120]
Dry-cured Iberian ham	OTA	2–160.9	Spain	[121]
Fermented meat products	OTA, CIT, AFB_1_	<0.05–7.83 <1.0–1.3 <1.0–3.0	Croatia	[100]
Traditional meat products	AFB_1_, OTA	<1.0–1.2 2.03–2.31	Croatia	[70,122]
Dry-fermented sausages	OTA, CPA	0.48 2.55–59.80	Croatia	[123,124]

**Table 3:** Biological control of fungal growth and mycotoxin production [137].

Agent	
Protein	Fungi
AFP	*Aspergillus giganteus*
Anafp	*Aspergillus niger*
AcAFP	*Aspergillus clavatus*
NFAP	*Neosartorya fischeri*
PAF	*Penicillium chrysogenum*
PgAFP	*Penicillium chrysogenum*
Pc-Arctin	*Penicillium chrysogenum*

**Table 4:** Overall presentation of common mycotoxins, their concentrations in meat and meat products, and related regulatory body for potential (future) legislation.

Mycotoxin	Regulatory Body	Total Daily Intake	Health Risk	Identified in	Concentration	Source
OTA	EFSA	120 ng/kg bw/week	Nephrotoxicity hepatotoxicity, immunotoxicity, neurotoxicity, teratogenicity, and carcinogenicity	Sausage	0.12 µg/kg	[3,100,154,155,156]
	Joint FAO/WHO	100 ng/kg bw */week		Dry-meat products	<LOQ- ≤ 7.83 µg/kg	
	Scientific Committee of Food (SCF) of the European Union	5 ng/kg bw */day		Ham	≤28.42 µg/kg	
				Salami	≤0.08 µg/kg	
				Pig muscle	≤0.04–0.06 µg/kg ≤0.14 µg/kg	
Aflatoxin B1	EFSA	4 µg/kg to 10 µg/kg for total aflatoxin	Genotoxicity, hepatotoxicity, immunotoxicity, teratogenicity, carcinogenicity	Sausage	1.5 µg/kg	[3,100,112,157,158,159,160,161]
	Joint FAO/WHO	Not more than 10 µg/kg for total aflatoxin of which aflatoxin B1 shall not be more than 5 µg/kg		Dry-meat products	<LOQ-3.0 µg/kg	
	Scientific Committee of Food (SCF) of the European Union	5–10 µg/kg for total aflatoxin		Ham	0.95–1.06 µg/kg	
				Pig muscle	0.46–0.74 µg/kg	
Aflatoxin B2	EFSA	4 µg/kg to 10 µg/kg for total aflatoxins	Hepatotoxicity, carcinogenicity, weak mutagenic effects	Sausage	3 µg/kg	[157,158,159,162,163]
	Joint FAO/WHO	Not more than 10 µg/kg for total aflatoxin of which aflatoxin B1 shall not be more than 5 µg/kg				
	Scientific Committee of Food (SCF) of the European Union	5–10 µg/kg for total aflatoxins				
Zearlenone	EFSA	0.25 µg/kg body weight	Reproductive toxicity, hepatotoxicity, immunotoxicity, genotoxicity and carcinogenicity, intestinal toxicity, endocrine disruption	Sausage	2.1–8.9 µg/kg	[101,164,165,166,167]
	Joint FAO/WHO	0.5 µg/kg bw *		Pig muscle	≤4.31 µg/kg	
Citrinin	EFSA	0.2 µg/kg bw * per day	Necrotic changes of parenchyma organs ephrotoxicity, gastrointestinal ailments, fetal malformations, and lymphoid tissue damage (additively, synergistically, or antagonistically to OTA)	Sausage	1.0 µg/kg	[100,102,105]
				Dry-meat products	<LOQ-1.3 µg/kg	
Sterigmatocystin	EFSA	Not established	Possible carcinogen, immunotoxic and immunomodulatory activity, together with mutagenic effect	Pig muscle	0.76–1.23 µg/kg	[111,112,168,169]
	Joint FAO/WHO					
	Scientific Committee of Food (SCF) of the European Union					
T-2 Toxin	EFSA	100 ng/kg bw * for T-2 toxins and HT-2 toxins	Anorexia, emesis, carcinogenicity, haematotoxicity, neurotoxicity and immunotoxicity	Pig muscle	0.0240–0.4515 µg/kg	[154,170,171,172,173,174]
	Joint FAO/WHO	25 ng/kg bw * for T-2, HT-2 and DAS, alone or in combination				
	Scientific Committee of Food (SCF) of the European Union	0.06 g/kg bw */day. for T-2 toxins and HT-2 toxins				

* bw—body weight; Joint FAO/WHO—Joint FAO/WHO Expert Committee on Food Additives (JECFA).

## Data Availability

Data are available upon request to the corresponding authors.
